# Finite-Time Topology Identification of Delayed Complex Dynamical Networks and Its Application

**DOI:** 10.34133/cbsystems.0092

**Published:** 2024-03-20

**Authors:** Yu Chen, Zhi-Wei Liu, Yuzhen Qin

**Affiliations:** ^1^ The School of Artificial Intelligence and Automation, Huazhong University of Science and Technology, Wuhan 430074, China.; ^2^ Department of Artificial Intelligence, Donders Institute for Brain, Cognition and Behaviour, Radboud University, Nijmegen 6525 GD, Netherlands.

## Abstract

To understand the functional behaviors of systems built on networks, it is essential to determine the uncertain topology of these networks. Traditional synchronization-based topology identification methods generally converge asymptotically or exponentially, resulting in their inability to give timely identification results. The finite-time stability theory is adopted in this paper with the aim of addressing the problem of fast identification of uncertain topology in networks. A novel finite-time topology observer is proposed to achieve finite-time topology identification and synchronization of general complex dynamical networks with time delay and second-order dynamical networks with time delay and nonlinear coupling. In addition, the proposed finite-time identification method is applied to power grids to address the problem of fast detection of line outages. Finally, 2 numerical experiments are provided to demonstrate the effectiveness and rapidity of the proposed finite-time identification method.

## Introduction

Various scenarios in the real world, such as the internet, transport networks, neural networks, and power grids, have typical complex network structures [1–4]. These networks consist of numerous interconnected nodes, with information flowing along the links between them. Consequently, the network’s connection structure profoundly influences its properties, including factors like scale-free characteristics and controllability [5–7]. However, the precise structure of these networks is frequently uncertain in practical situations. Thus, inferring the inherent topology of complex networks becomes a prerequisite for comprehending and explaining the evolutionary mechanisms and functional behaviors of the systems operating within them [8]. Accurate topology identification is critical for various applications such as network control, optimization, and security.

Over the last decade, several innovative methods have been proposed to tackle the challenges of topology identification in complex networks. These methods are usually classified into the following categories.

1. Optimization methods: Modeling the complex network topology identification problem as an optimization problem and solving it using optimization algorithms [9–12]. Recent research aims to simplify network topology identification by treating it as a sparse signal reconstruction problem. This approach leverages the sparse nature of complex network topology, transforming topology identification into a convex optimization problem, which is then solved using compressed sensing algorithms. A theoretical framework based on compressed sensing and regularization was proposed in [11] to identify one layer or multilayer networks. From the perspective of Bayesian statistics, a new framework developed for network structure reconstruction was proposed in [12], which utilized sparse Bayesian learning based on evolutionary game data. The aforementioned algorithms [9–12] require data accumulation over a period of time for topology identification of complex networks. This implies that the complex network topology structure cannot change during the data sampling period. In contrast, the proposed observer-based algorithm does not require data collection and can quickly identify changes in the topology edge weights. Furthermore, these optimization-based methods are not all capable of achieving 100% identification accuracy, while the observer-based methods can identify all edge weights in the topology once the observer converges.
2. Machine learning methods: Utilizing supervised or unsupervised learning algorithms to identify patterns and predict network topology [13–16]. Xu et al. [14] transformed the problem of complex network reconstruction into a regression problem and utilized the variational Bayesian statistical method to solve it. The identification accuracy of this method decreases as the complexity of the network structure rises. A sparse dynamical Boltzmann machine was developed in [15] to identify the structure of complex networks hosting binary dynamical processes, which was developed using compressed sensing and a clustering algorithm. A statistical inference-based method was proposed in [16], which utilized the expectation maximization algorithm based solely on binary data to reconstruct complex network structures. When these methods [15,16] reconstruct network structures with binary state dynamics, they rely on 2 assumptions related to node activity states and transition probabilities, which reduces the accuracy of topology identification and might hinder their application.
3. Synchronization-based methods: On the basis of graph theory, the response network is designed to synchronize the original drive network for topology identification [17–21].

Most of the current methods used to identify complex network topology are based on synchronization. In [19], Wang et al. investigated the problem of asymptotic synchronization-based topology identification and parameter identification of 2-layer complex dynamical networks. In [8], Wu et al. investigated the issue of dynamic identification of complex networks in the presence of stochastic perturbations.

Both Liu et al. [20] and Zhu et al. [21] have proposed an auxiliary network consisting of isolated nodes to achieve outer synchronization and topology identification of complex networks, whereas the design of isolated nodes is different between the 2 methods. The identification methods [20,21] achieve synchronization and topology identification between the regulated network and the auxiliary network by adding controllers to the original network. However, if the original network cannot be controlled and can only be observed, these 2 methods become ineffective. Moreover, these synchronization-based methods can only achieve asymptotic convergence, and the speed of identifying the topology needs to be improved.

Furthermore, the complex network topology identification approach can also be used to estimate the admittance values of transmission lines in power grids. In [22], the power grid is conceptualized as a complex network with unspecified parameters, and an innovative line-outage detection algorithm is introduced through the use of an adaptive virtual observer. In this model, the transmission lines connecting the buses serve as network edges, while the line admittance values function as edge weights. Once a line outage occur between 2 connected buses, the line’s admittance value drops to zero. As the virtual observer reaches a stable state, the monitored admittance values align with the actual ones. The algorithm can thus pinpoint the location of line outages by tracking whether observed admittance values converge to zero. Expanding upon [22], the study [23] takes into account the influence of load stochastic perturbations, which can introduce dynamic disturbances into the power grid, as discussed in [24]. Although these algorithms can accurately identify the location of outage lines in the power grid, they use the adaptive asymptotic convergence observation method, which has the drawback of slow convergence speed, necessitating further improvements in detection speed.

Some progress has been made in finite-time topology identification of complex dynamical networks [25–27]. These methods rely on the difference between the true values of the edge weights in the drive network (the quantities to be identified) and the estimated values of the edge weights in the response network, which is contrary to the original intention of performing topology identification. This also limits the application of the identification methods in power grid topology identification and line-outage detection.

Motivated by the above analysis, this paper proposes a novel finite-time identification method for delayed complex networks with linear coupling or nonlinear coupling and applies it to the power grid for anomaly detection. The finite-time method allows for the observation of the topology and its changes within a finite time, enabling the detection of line outages in finite time. By utilizing finite-time stability theory, we establish conditions for achieving finite-time convergence and a relationship between convergence time and the initial error system values. Main contributions of this paper are listed as follows:

1. The proposed finite-time identification method achieves finite-time topology identification and line-outage detection by utilizing information from itself and neighbors rather than the difference between the true values of the edge weights in the drive network and the estimated values of the edge weights in the response network, making it applicable for uncertain topology identification scenarios.
2. Applying the finite-time topology identification method to the second-order dynamical network with nonlinear coupling and time delay and presenting a practical application case of the power grid based on a second-order Kuramoto model. A distributed finite-time observer is designed for the power grid to achieve fast and accurate location of outages.

**Notations:** *ℝ^n^* denotes the *n*-dimensional real Euclidean space, *ℝ*^*n*×*m*^ is the set of all *n* × *m* real matrices. (*x*_1_, *x*_2_, …, *x_n_*)^⊤^ is denoted by *x*. The spectral norm of *x* is denoted as $\left\|x\right\|={\left(\lambda_{\text{max}}\left(x^{⊤}x\right)\right)}^{\frac{1}{2}}$, where the superscript ⊤ denotes transposition. The signnum function is denoted by *sgn*(. ), which is given below:

$$\text{sgn}\left(x\right)=\left\{\begin{array}{c} -1,\text{if}\;x<0,\\ 0,\text{if}\;x=0,\\ 1,\text{if}\;x>0. \end{array}\right.$$

The remainder of this paper is organized as follows. The “Materials” section outlines some useful assumptions and lemmas. The “Finite-Time Topology Identification Method” section explores topology identification of complex networks with time delay and gives a rigorous mathematical proof. In the “Application of the Finite-Time Observer to Power Grids” section, the proposed finite-time identification approach is applied to second-order dynamical networks with time delay and nonlinear coupling, which is also be applied to the field of line-outage detection in power grids and a finite-time line-outage detection algorithm is proposed. The “Results and Discussion” section provides 2 numerical experiments to confirm the effectiveness of the proposed method. The comparison experiments show that this method is superior to the adaptive method. The main work of this paper is summarized in the “Conclusion” section.

## Materials

To achieve the objective of this paper, some necessary mathematical preliminaries and problem formulations are presented in this section, including finite-time theory, observation framework, and so on.

### Mathematical preliminaries

Before designing the finite-time topology identification method, some useful assumptions and lemmas are introduced below.

**Assumption 2.1**. Suppose that a nonlinear function *f_i_*(.) satisfies the local Lipschitz condition, then for any 2 vectors *x_i_*, *y_i_* ∈ *ℝ^n^*, there exists a non-negative constant *L_i_* satisfying

$$∥f_{i}\left(x_{i}\right)-f_{i}\left(y_{i}\right)∥\leq L_{i}∥x_{i}-y_{i}∥,i=1,2,\ldots ,N.$$

**Assumption 2.2**. Suppose that ${\left\{Hx_{i}(t)\right\}}_{i=1}^{N}$ are linearly independent on the orbit ${\left\{x_{i}(t)\right\}}_{i=1}^{N}$ of the synchronization manifold ${\left\{x_{i}(t)=y_{i}(t)\right\}}_{i=1}^{N}$

**Assumption 2.3**. Suppose that time-varying delay *τ*(*t*) is differentiable and satisfies $\overset{\cdot }{\tau }\left(t\right)\leq \beta <1$*,* where *β* is a constant.

**Lemma 2.1**. [28] For any vectors *x*, *z* ∈ *ℝ^n^* and any positive definite matrix *P* ∈ *ℝ*^*n*×*n*^, the following inequality holds:

$$2x^{⊤}z\leq x^{⊤}\mathrm{Px}+z^{⊤}P^{-1}z$$

**Lemma 2.2**. [29] For $p_{i}\geq 0$, i=1,2,⋯,N, and 0<η<2 is a real number, there is

$$∥p_{1}{∥}^{\eta }+\cdots +∥p_{n}{∥}^{\eta }\geq {\left(∥p_{1}{∥}^{2}+\cdots +∥p_{n}{∥}^{2}\right)}^{\frac{\eta }{2}}$$

**Lemma 2.3**. [30] If a continuous non-negative definite function *V*(*t*) satisfies

$$\overset{\cdot }{V}\left(t\right)\leq -\alpha V^{\eta }\left(t\right),\forall t\geq t_{0},V\left(t_{0}\right)\geq 0$$

where *α* > 0, 0 < *η* < 1. Then, *V*(*t*) meets

$$\begin{array}{c} V^{1-\eta }\left(t\right)\leq V^{1-\eta }\left(t_{0}\right)-\alpha \left(1-\xi \right)\left(t-t_{0}\right),t_{0}\leq t\leq t^{*} \end{array}$$

and *V*(*t*) = 0, *t* ≥ t^*^, where

$$t^{*}=t_{0}+\frac{1}{\alpha \left(1-\eta \right)}V^{1-\eta }\left(t_{0}\right)$$

### Problem formulation

**Fig. 1.**
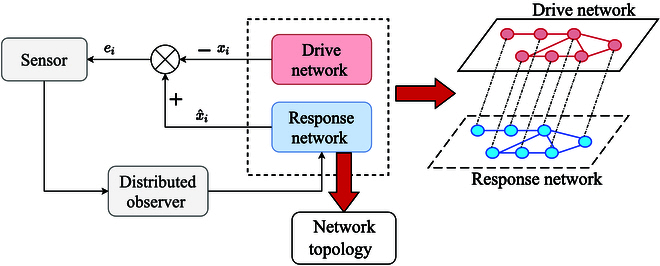
Distributed observation framework of complex dynamical networks.

In reality, the exact topology structure of a complex network is usually uncertain, making its estimation crucial in various scenarios. We designed a response network to observe the original drive network, as illustrated in Fig. 1, where *x_i_*, ${\hat{x}}_{i}$, and *e_i_* denote the state information, the observed state information, and state error information for node *i*, respectively.

While we can access the node states of the drive network, the edge weights of its topology remain uncertain. There have been many studies to design an adaptive observation network to identify the topology of the driving network by exploiting the state observation error, but the convergence of the adaptive observer is slow and cannot identify the network topology in a short time. Therefore, our aim is to design an observer with faster recognition speed. The node states of the drive network can be obtained, while the edge weights of the topology are unknown and need to be observed. We primarily use the state observation errors to design a finite-time observation network, achieving rapid identification of the drive network topology.

## Finite-Time Topology Identification Method

In this section, a finite-time method is proposed to estimate the topology of complex networks, which is a departure from the existing finite-time topology identification methods ([25–27]) in that it does not rely on the difference between the true values of the edge weights in the driving network and the estimated values of the edge weights in the responding network and that it can achieve convergence in a finite time based only on the information of the node state errors and the observation information.

### Design of finite-time identification method

Consider a general complex dynamical network consisting of *N* nodes, which is given below.

$$\begin{array}{c} {\overset{\cdot }{x}}_{i}\left(t\right)=f_{i}\left(t,x_{i}\left(t\right)\right)+\sum_{j=1,j\neq i}^{N}a_{\mathrm{ij}}H\left(x_{j}\left(t-\tau \left(t\right)\right)-x_{i}\left(t\right)\right),\\ \;i=1,2,\ldots ,N, \end{array}$$ (1)

where *x_i_* = (*x*_*i*1_, *x*_*i*2_, …, *x_in_*)^⊤^ ∈ *ℝ^n^* represents the state vector of node *i* and *f*(·) : *ℝ^n^* → *ℝ^n^* governs the self-dynamics of node *i*. *τ*(*t*) denotes the time delay caused by signal communication, which is non-negative and can be either time-varying or constant.

The interaction pattern between nodes is determined by the inner coupling matrix *H* ∈ *ℝ*^*n*×*n*^. The weight of the edge (*i*, *j*) in the complex network is described by *a_ij_*. Specifically, *a_ij_* > 0 if there exists an edge from node *j* to node *i*, and *a_ij_* = 0 otherwise. For the diagonal elements, set $a_{\mathrm{ii}}=-\;\sum_{j=1,j\neq i}^{N}a_{\mathrm{ij}}$ for *i* = 1,2, …, *N*. The adjacency matrix of the network is denoted by *A* = [*a_ij_*] ∈ *ℝ*^*N*×*N*^.

Considering that not all nodes in a complex network are interconnected, we propose a distributed computation for each node. This means each node only exchanges information with those it is directly connected to, which significantly reduces the computational burden. The drive network (Eq. 1) can be modified as

$$\begin{array}{c} {\dot{x}}_{i}\left(t\right)=f_{i}\left(t,x_{i}\left(t\right)\right)+\sum_{j\in N_{i}}a_{\mathrm{ij}}H\left(x_{j}\left(t-\tau \left(t\right)\right)-x_{i}\left(t\right)\right),\\ \; \end{array}$$ (2)

where $N_{i}$ denotes the set of nodes connected to node *i*.

To identify the uncertain topology *A* of the general complex network, it is common to construct a response network to synchronize it, given by:

$$\begin{array}{c} {\dot{\hat{x}}}_{i}\left(t\right)=f_{i}\left(t,{\hat{x}}_{i}\left(t\right)\right)+\sum_{j\in N_{i}}{\hat{a}}_{\mathrm{ij}}H\left({\hat{x}}_{j}\left(t-\tau \left(t\right)\right)-{\hat{x}}_{i}\left(t\right)\right)+u_{i}\left(t\right),\\ \; \end{array}$$ (3)

where ${\hat{x}}_{i}={\left({\hat{x}}_{i1},{\hat{x}}_{i2},\ldots ,{\hat{x}}_{\text{in}}\right)}^{⊤}\in {\mathbb{R}}^{n}$ is the state vector of node *i* in the response network, ${\hat{a}}_{\mathrm{ij}}$ for *j* ≠ *i* are the estimated values of *a_ij_*. *u_i_*(*t*) and ${\hat{a}}_{\mathrm{ij}}\left(t\right)$ are given by

$$\begin{array}{c} u_{i}\left(t\right)=-d_{i}\left(t\right)\left({\hat{x}}_{i}\left(t\right)-x_{i}\left(t\right)\right)-\alpha \text{sgn}\left({\hat{x}}_{i}\left(t\right)-x_{i}\left(t\right)\right), \end{array}$$ (4a)

$$\begin{array}{c} \begin{array}{l} {\overset{\cdot }{\hat{a}}}_{\mathrm{ij}}\left(t\right)=-\sigma_{\mathrm{ij}}{\left({\hat{x}}_{i}\left(t\right)-x_{i}\left(t\right)\right)}^{⊤}H\left({\hat{x}}_{j}\left(t-\tau \left(t\right)\right)-{\hat{x}}_{i}\left(t\right)\right)\\ \;-\sigma_{\mathrm{ij}}\;\text{sgn}{\left({\hat{x}}_{i}\left(t\right)-x_{i}\left(t\right)\right)}^{⊤}H\left({\hat{x}}_{j}\left(t-\tau \left(t\right)\right)-{\hat{x}}_{i}\left(t\right)\right), \end{array} \end{array}$$ (4b)

and

$$\begin{array}{c} \left\{\begin{array}{c} {\dot{d}}_{i}\left(t\right)=k_{i}{\left({\hat{x}}_{i}\left(t\right)-x_{i}\left(t\right)\right)}^{⊤}\left({\hat{x}}_{i}\left(t\right)-x_{i}\left(t\right)\right)-\alpha \sqrt{k_{i}}\mathrm{sgn}(d_{i}\left(t\right)-d^{*})\\ \;-\frac{k_{i}(d_{i}\left(t\right)-d^{*}}{{\left(d_{i}\left(t\right)-d^{*}\right)}^{2}}\left(\Gamma_{1}+\Gamma_{2}+\Gamma_{3}\right),\text{if }{\text{d}}_{i}\text{(}\text{t}\text{)≠}{\text{d}}^{*}\text{,}\;\\ \;{\dot{d}}_{i}\left(t\right)=k_{i}{\left({\hat{x}}_{i}\left(t\right)-x_{i}\left(t\right)\right)}^{⊤}\left({\hat{x}}_{i}\left(t\right)-x_{i}\left(t\right)\right),\text{if }{\text{d}}_{i}\text{(}\text{t}\text{)=}{\text{d}}^{*}\text{,}\; \end{array}\right. \end{array}$$ (4c)

where *i* = 1, …, *n*, $j\in N_{i},d^{*}$ is a sufficiently large positive number, *k_i_*, *σ_ij_*, and *α* are positive constants, *α* > 0. Γ_1_, Γ_2_, and Γ_3_ are given by:

$$\Gamma_{1}=\sum_{j\in N_{i}}{\left\|(|{\hat{a}}_{\mathrm{ij}}\left(t\right)|+\xi )H\left({\hat{x}}_{j}\left(t-\tau \left(t\right)\right)-{\hat{x}}_{i}\left(t\right)\right)\right\|}_{1},$$ (5)

$$\Gamma 2=\alpha \sum_{j\in N_{i}}\left(\frac{|{\hat{a}}_{\mathrm{ij}}\left(t\right)|+\xi }{\sqrt{\sigma_{\mathrm{ij}}}}\right),$$ (6)

and

$$\begin{array}{c} \Gamma_{3}=\frac{\alpha }{1-\beta }{\left(\;\int_{t-\tau (t)}^{t}\sum_{i=1}^{N}e_{i}^{⊤}\left(s\right)e_{i}\left(s\right)ds\;\right)}^{\frac{1}{2}}, \end{array}$$ (7)

respectively, where *ξ* is a positive constant, given by:

$$\text{max}|a_{\mathrm{ij}}|\leq \xi ,i=1,2,\ldots ,N,j\in N_{i}.$$ (8)

Denoting $e_{i}\left(t\right)={\hat{x}}_{i}\left(t\right)-x_{i}\left(t\right)$ and ${\tilde{a}}_{\mathrm{ij}}={\hat{a}}_{\mathrm{ij}}-a_{\mathrm{ij}}$ , we can obtain the following the error system:

$$\begin{array}{c} {\dot{e}}_{i}\left(t\right)=f_{i}\left(t,{\hat{x}}_{i}\left(t\right)\right)-f_{i}\left(t,x_{i}\left(t\right)\right)-d_{i}\left(t\right)e_{i}\left(t\right)-\alpha \text{sgn}\left(e_{i}\left(t\right)\right)\\ \;+\sum_{j\in N_{i}}{\tilde{a}}_{\mathrm{ij}}H\left({\hat{x}}_{j}\left(t-\tau \left(t\right)\right)-{\hat{x}}_{i}\left(t\right)\right)+\sum_{j\in N_{i}}a_{\mathrm{ij}}H\left(e_{j}\left(t-\tau \left(t\right)\right)-e_{i}\left(t\right)\right). \end{array}$$ (9)

**Remark 3.1**. The existing finite-time topology identification methods [25–27] heavily rely on the difference between the the true values of the edge weights in the drive network and the estimated values of the edge weights in the response network, which is not obtainable in the field of complex networks topology identification. Conversely, the proposed finite-time observation method eliminates the need for the difference between the true values of edge weights and their observed values and enables rapid identification of the network topology.

### Convergence analysis

**Theorem 3.1**. Suppose that Assumptions 2.1 to 2.3 hold. Then, the uncertain coupling configuration matrix *A* of the complex network (Eq. 1) can be identified by the proposed observer (Eqs. 3 and 4a to 4c) in finite-time *t_c_* under any conditions, where *t_c_* is the upper bound of the convergence time, given by:

$$t_{c}=\frac{\sqrt{2V_{c}^{1/2}\left(0\right)}}{\alpha }$$ (10)

and *V_c_*(0) is given by:

Vc(0)=12∑i=1Nei⊤(0)ei(0)+12∑i=1N∑j∈Ni1σija˜ij2(0)+12∑i=1N1ki(di(0)−d*)2+12(1−β)∫−τ(0)0∑i=1Nei⊤(s)ei(s)ds. (11)

**Proof:** Construct a Lyapunov function *V_c_* as follows:

Vc(t)=12∑i=1Nei⊤(t)ei(t)+12∑i=1N∑j∈Ni1σija˜ij2+12∑i=1N1ki(di(t)−d*)2+12(1−β)∫t−τ(t)t∑i=1Nei⊤(s)ei(s)ds. (12)

Taking the derivative of *V_c_* along the trajectory of Eq. 9 yields

$$\begin{array}{c} \begin{array}{l} {\overset{\cdot }{V}}_{c}\left(t\right)=\sum_{i=1}^{N}e_{i}^{⊤}\left(t\right){\overset{\cdot }{e}}_{i}\left(t\right)+\sum_{i=1}^{N}\sum_{j\in N_{i}}\frac{1}{\sigma_{\mathrm{ij}}}{\tilde{a}}_{\mathrm{ij}}{\overset{\cdot }{\tilde{a}}}_{\mathrm{ij}}+\sum_{i=1}^{N}\frac{1}{k_{i}}\left(d_{i}\left(t\right)-d^{*}\right){\overset{\cdot }{d}}_{i}\left(t\right)\\ \;+\frac{1}{2\left(1-\beta \right)}\sum_{i=1}^{N}e_{i}^{⊤}\left(t\right)e_{i}\left(t\right)-\frac{1-\overset{\cdot }{\tau }\left(t\right)}{2\left(1-\beta \right)}\sum_{i=1}^{N}e_{i}^{⊤}\left(t-\tau \left(t\right)\right)e_{i}\left(t-\tau \left(t\right)\right)\\ \;=\sum_{i=1}^{N}e_{i}^{⊤}\left(t\right)\;\left(f_{i}\left(t,{\hat{x}}_{i}\left(t\right)\right)-f_{i}\left(t,x_{i}\left(t\right)\right)\right)-d^{*}\;\sum_{i=1}^{N}e_{i}^{⊤}\left(t\right)e_{i}\left(t\right)\\ \;+\sum_{i=1}^{N}\sum_{j\in N_{i}}e_{i}^{⊤}\left(t\right)a_{\mathrm{ij}}H\left(e_{j}\left(t-\tau \left(t\right)\right)-e_{i}\left(t\right)\right)-\sum_{i=1}^{N}\\ \;\sum_{j\in N_{i}}{\tilde{a}}_{\mathrm{ij}}\text{sgn}\left(e_{i}^{⊤}\left(t\right)\right)H\left({\hat{x}}_{j}\left(t-\tau \left(t\right)\right)-{\hat{x}}_{i}\left(t\right)\right)\\ \;-\sum_{i=1}^{N}\sum_{j\in N_{i}}{\left\|\left(|{\hat{a}}_{\mathrm{ij}}|+\xi \right)H\left({\hat{x}}_{j}\left(t-\tau \left(t\right)\right)-{\hat{x}}_{i}\left(t\right)\right)\right\|}_{1}-\alpha \sum_{i=1}^{N}\frac{1}{1-\beta }{\left(\int_{t-\tau \left(t\right)}^{t},e_{i}^{⊤},\left(s\right),e_{i},\left(s\right),\mathrm{ds}\right)}^{\frac{1}{2}}\\ \;-\alpha \;\sum_{i=1}^{N}\left|\frac{1}{\sqrt{k_{i}}}\left(d_{i}\left(t\right)-d^{*}\right)\right|-\alpha \;\sum_{i=1}^{N}\sum_{j\in N_{i}}\left(\frac{|{\hat{a}}_{\mathrm{ij}}|+\xi }{\sqrt{\sigma_{\mathrm{ij}}}}\right)\\ \;+\frac{1}{2\left(1-\beta \right)}\sum_{i=1}^{N}e_{i}^{⊤}\left(t\right)e_{i}\left(t\right)-\frac{1-\overset{\cdot }{\tau }\left(t\right)}{2\left(1-\beta \right)}\sum_{i=1}^{N}e_{i}^{⊤}\left(t-\tau \left(t\right)\right)e_{i}\left(t-\tau \left(t\right)\right). \end{array} \end{array}$$ (13)

Denote $\begin{array}{c} e\left(t\right)={\left(e_{1}^{⊤}(t),e_{2}^{⊤}(t),\ldots e_{N}^{⊤})\right)}^{⊤}\in {\mathbb{R}}^{nN},L\;=\underset{1\leq i\leq N}{\text{max}}\left\{L_{i}\right\} \end{array}$, and *P* = *A* ⊗ *H*, where ⊗ denotes the Kronecker tensor product. Then according to Assumption 2.1, we can obtain

$$\begin{array}{c} \begin{array}{l} {\overset{\cdot }{V}}_{c}\leq \left(L-d^{*}+\frac{1}{2\left(1-\beta \right)}\right)e^{⊤}\left(t\right)e\left(t\right)+e^{⊤}\left(t\right)\mathrm{Pe}\left(t-\tau \right)\\ \;+\sum_{i=1}^{N}\sum_{j\in N_{i}}{\left\|{\tilde{a}}_{\mathrm{ij}}H\left({\hat{x}}_{j}\left(t-\tau \right)-{\hat{x}}_{i}\left(t\right)\right)\right\|}_{1}-\sum_{i=1}^{N}\sum_{j\in N_{i}}{\left\|(|{\hat{a}}_{\mathrm{ij}}|+\xi )H({\hat{x}}_{j}\left(t-\tau \right)-{\hat{x}}_{i}\left(t\right)\right\|}_{1}\\ \;-\alpha \;\sum_{i=1}^{N}\|e_{i}\left(t\right)\|-\alpha \;\sum_{i=1}^{N}\left|\frac{1}{\sqrt{k_{i}}},\left(d_{i}\left(t\right)-d^{*}\right)\right|-\alpha \;\sum_{i=1}^{N}\sum_{j\in N_{i}}\left(\frac{|{\hat{a}}_{\mathrm{ij}}|+\xi }{\sqrt{\sigma_{\mathrm{ij}}}}\right)\\ \;-\alpha \;\sum_{i=1}^{N}\frac{1}{1-\beta }\left(\int_{t-\tau \left(t\right)}^{t}e_{i}^{⊤}\left(s\right)e_{i}\left(s\right)\mathrm{ds}\right)-\frac{1-\overset{\cdot }{\tau }\left(t\right)}{2\left(1-\beta \right)}e^{⊤}\left(t-\tau \left(t\right)\right)e\left(t-\tau \left(t\right)\right). \end{array} \end{array}$$ (14)

According to Eq. 8, we can obtain

$$|{\hat{a}}_{\mathrm{ij}}|+\zeta \geq |{\hat{a}}_{\mathrm{ij}}|+|a_{\mathrm{ij}}|\geq |{\hat{a}}_{\mathrm{ij}}-a_{\mathrm{ij}}|=|{\tilde{a}}_{\mathrm{ij}}|,$$ (15)

and then Eq. 14 can be written as

$$\begin{array}{c} \begin{array}{l} {\overset{\cdot }{V}}_{c}\leq \left(L-d^{*}+\frac{1}{2\left(1-\beta \right)}\right)e^{⊤}\left(t\right)e\left(t\right)+e^{⊤}\left(t\right)\mathrm{Pe}\left(t-\tau \right)\\ \;-\alpha \;\sum_{i=1}^{N}\|e_{i}\left(t\right)\|-\alpha \;\sum_{i=1}^{N}\left|\frac{\left(d_{i}\left(t\right)-d^{*}\right)}{\sqrt{k_{i}}}\right|-\alpha \;\sum_{i=1}^{N}\sum_{j\in N_{i}}\left(\frac{|{\hat{a}}_{\mathrm{ij}}|+\xi }{\sqrt{\sigma_{\mathrm{ij}}}}\right)\\ \;-\alpha \;\sum_{i=1}^{N}\frac{1}{1-\beta }\left(\int_{t-\tau \left(t\right)}^{t}e_{i}^{⊤}\left(s\right)e_{i}\left(s\right)\mathrm{ds}\right)-\frac{1-\overset{\cdot }{\tau }\left(t\right)}{2\left(1-\beta \right)}e^{⊤}\left(t-\tau \left(t\right)\right)e\left(t-\tau \left(t\right)\right). \end{array} \end{array}$$ (16)

According to Lemma 2.1, it can be derived that

$$\begin{array}{c} e^{⊤}\left(t\right)\mathrm{Pe}\left(t-\tau \right)\leq \frac{1}{2}\left[e^{⊤}\left(t\right){\mathrm{PP}}^{⊤}e\left(t\right)+e^{⊤}\left(t-\tau \right)e\left(t-\tau \right)\right], \end{array}$$ (17)

and according to Assumption 2.3, one has

$$\frac{1-\overset{\cdot }{\tau }\left(t\right)}{2\left(1-\beta \right)}\geq \frac{1}{2},$$ (18)

and then Eq. 16 can be written as

$$\begin{array}{c} \begin{array}{l} {\overset{\cdot }{V}}_{c}\leq \left(L-d^{*}+\frac{1}{2\left(1-\beta \right)}\right)e^{⊤}\left(t\right)e\left(t\right)+e^{⊤}\left(t\right)\mathrm{Pe}\left(t-\tau \right)\\ \;+\left(\frac{1}{2}-\frac{1-\overset{\cdot }{\tau }\left(t\right)}{2\left(1-\beta \right)}\right)e^{⊤}\left(t-\tau \left(t\right)\right)e\left(t-\tau \left(t\right)\right)-\alpha \;\sum_{i=1}^{N}\|e_{i}\left(t\right)\|-\alpha \;\sum_{i=1}^{N}\sum_{j\in N_{i}}\left(\frac{|{\hat{a}}_{\mathrm{ij}}|+\xi }{\sqrt{\sigma_{\mathrm{ij}}}}\right)\\ \;-\alpha \;\sum_{i=1}^{N}\left|\frac{1}{\sqrt{k_{i}}}(d_{i}\left(t\right)-d^{*}\right|-\alpha \;\sum_{i=1}^{N}\frac{1}{2\left(1-\beta \right)}{\left(\int_{t-\tau \left(t\right)}^{t}e_{i}^{⊤}\left(s\right)e_{i}\left(s\right)\mathrm{ds})\right)}^{\frac{1}{2}}\\ \;\leq \left(L-d^{*}+\frac{1}{2\left(1-\beta \right)}+\frac{1}{2}\lambda_{\text{max}}\left({\mathrm{PP}}^{⊤}\right)\right)e^{⊤}\left(t\right)e\left(t\right)-\alpha \;\sum_{i=1}^{N}\frac{1}{\left(1-\beta \right)}{\left(\int_{t-\tau \left(t\right)}^{t}e_{i}^{⊤}\left(s\right)e_{i}\left(s\right)\mathrm{ds}\right)}^{\frac{1}{2}}\\ \;-\alpha \;\sum_{i=1}^{N}\|e_{i}\left(t\right)\|-\alpha \;\sum_{i=1}^{N}\left|\frac{1}{\sqrt{k_{i}}}\left(d_{i}\left(t\right)-d^{*}\right)\right|-\alpha \;\sum_{i=1}^{N}\sum_{j\in N_{i}}\left(\frac{|{\hat{a}}_{\mathrm{ij}}|+\xi }{\sqrt{\sigma_{\mathrm{ij}}}}\right). \end{array} \end{array}$$ (19)

Let $d^{*}$ be a constant larger than $L+\frac{1}{2\left(1-\beta \right)}+\frac{1}{2}\lambda_{\text{max}}\left(PP^{⊤}\right)$, and then according to Lemma 2.2, we can obtain

$$\begin{array}{c} \begin{array}{l} {\overset{\cdot }{V}}_{c}\leq -\sqrt{2}\alpha \;[\frac{1}{2}\sum_{i=1}^{N}e_{i}^{⊤}\left(t\right)e_{i}\left(t\right)+-\frac{1}{2}\sum_{i=1}^{N}\sum_{j\in N_{i}}\frac{1}{\sigma_{\mathrm{ij}}}{\tilde{a}}_{\mathrm{ij}}^{2}\\ \;{\;+\frac{1}{2}\sum_{i=1}^{N}\frac{1}{k_{i}}{\left(d_{i}\left(t\right)-d^{*}\right)}^{2}+\frac{1}{2\left(1-\beta \right)}\int_{t-\tau \left(t\right)}^{t}e_{i}^{⊤}\left(s\right)e_{i}\left(s\right)\mathrm{ds}]}^{\frac{1}{2}}\\ \;\leq -\sqrt{2}\alpha V_{c}^{1/2} \end{array} \end{array}$$ (20)

Finally, according to Lemma 2.3, when $t\geq t_{c}=\frac{\sqrt{2}V_{c}^{1/2}\left(0\right)}{\alpha }$, *V_c_*(*t*) = 0, the drive network (Eq. 2) and the response network (Eq. 3) achieve finite-time synchronization, and the uncertain parameters in the drive network can be correctly identified. From Eq. 12, it can obtained that $\underset{t\to t_{c}}{\text{lim}}\|e_{i}\left(t\right)\|=0,\underset{t\to t_{c}}{\text{lim}}|{\tilde{a}}_{\mathrm{ij}}\left(t\right)|=0$, and $\underset{t\to t_{c}}{\text{lim}}|d_{i}\left(t\right)-d^{*}|=0$.

**Remark 3.2**. The key to ensuring successful topology identification lies in the linear independence condition stated in Assumption 2.2. This condition guarantees that when synchronization is achieved within the uncertain network, commonly referred to as “inner synchronization”, the topology becomes unidentifiable. In other words, for successful identification, the dynamics of each node in the uncertain network should not be dependent on the dynamics of the other nodes.

**Remark 3.3**. Appropriate parameters such as *α*, *σ_ij_*, *k_i_*, and $d^{*}$ can be designed to allow the proposed observer to achieve a relatively rapid convergence speed.

## Application of the Finite-Time Observer to Power Grids

Power grids can be viewed as complex dynamical networks consisting of multiple Kuramoto oscillators. Specifically, the model of generator nodes can be regarded as second-order Kuramoto oscillator network, while the model of load nodes can be regarded as general Kuramoto oscillator network. The power grid model exhibits certain differences from general complex network models. In complex networks, adjacent buses are linked through linear coupling, whereas in power grids, adjacent nodes are linked through nonlinear coupling.

In this section, a general finite-time topology identification method for dynamical networks with nonlinear coupling is given, and then the method is applied to power grids to design a novel distributed multiple line-outage detection method.

### Topology identification of networks with nonlinear coupling

Consider a second-order dynamical network with nonlinear coupling consisting of *N* nodes, which is given below.

$$\begin{array}{c} \left\{\begin{array}{c} {\dot{w}}_{i}\left(t\right)=v_{i}\left(t\right),\\ {\dot{v}}_{i}\left(t\right)=f\left(t,v_{i},(t)\right)+\sum_{j\in N_{i}}a_{\mathrm{ij}}\phi_{\mathrm{ij}}\left(w_{j}\left(t-\tau \left(t\right)\right)-w_{i}\left(t\right)\right),i=1,2,\ldots ,N, \end{array}\right. \end{array}$$ (21)

where *w_i_*(*t*) = (*w*_*i*1_(*t*), *w*_*i*2_(*t*), …, *w_in_*(*t*))^⊤^ and *v_i_*(*t*) = (*v*_*i*1_(*t*), *v*_*i*2_(*t*), …, *v_in_*(*t*))^⊤^ represent the state and frequency of node *i*, respectively. *f*(·) : *ℝ^n^* → *ℝ^n^* governs the self-dynamics of node *i*.*ϕ_ij_*(*w_j_*(*t* − *τ*(*t*)) − *w_i_*(*t*)) = [*ϕ_ij_*(*w*_*j*1_(*t* − *τ*(*t*)) − *w*_*i*1_(*t*)), …, *ϕ_ij_*(*w_jn_*(*t* − *τ*(*t*)) − *w_in_*(*t*))]^⊤^ denotes the nonlinear coupling between nodes *i* and *j*.

The response network is given by:

$$\begin{array}{c} \left\{\begin{array}{c} {\dot{\hat{w}}}_{i}\left(t\right)={\hat{v}}_{i}\left(t\right)+u_{i}^{w}\left(t\right),\\ {\dot{\hat{v}}}_{i}\left(t\right)=f\left(t,{\hat{v}}_{i},(t)\right)+\sum_{j\in N_{i}}{\hat{a}}_{\mathrm{ij}}\phi_{\mathrm{ij}}\left({\hat{w}}_{j}\left(t-\tau \left(t\right)\right)-{\hat{w}}_{i}\left(t\right)\right)+u_{i}^{v}\left(t\right),i=1,2,\ldots ,N, \end{array}\right. \end{array}$$ (22)

where ${\hat{w}}_{i}\left(t\right)={\left({\hat{w}}_{i1}\left(t\right),{\hat{w}}_{i2}\left(t\right),\ldots ,{\hat{w}}_{\text{in}}\left(t\right)\right)}^{⊤}$ and ${\hat{v}}_{i}\left(t\right)=({\hat{v}}_{i1}\left(t\right),$ ${\hat{v}}_{i2}\left(t\right),\ldots ,{\hat{v}}_{\text{in}}\left(t\right){)}^{⊤}$ denote the state and frequency of node *i* in the response network. *u_i_^w^*(*t*), *u_i_^v^*(*t*), and ${\hat{a}}_{\mathrm{ij}}\left(t\right)$ are given by

$$u_{i}^{w}\left(t\right)=-d_{i}^{w}\left(t\right)\left({\hat{w}}_{i}\left(t\right)-w_{i}\left(t\right)\right)-\alpha \text{sgn}\left({\hat{w}}_{i}\left(t\right)-w_{i}\left(t\right)\right)$$ (23a)

$$u_{i}^{v}\left(t\right)=-d_{i}^{v}\left(t\right)\left({\hat{v}}_{i}\left(t\right)-v_{i}\left(t\right)\right)-\alpha \text{sgn}\left({\hat{v}}_{i}\left(t\right)-v_{i}\left(t\right)\right)$$ (23b)

$$\begin{array}{c} \begin{array}{c} {\overset{\cdot }{\hat{a}}}_{\mathrm{ij}}\left(t\right)=-\sigma_{\mathrm{ij}}{\left({\hat{v}}_{i}\left(t\right)-v_{i}\left(t\right)\right)}^{⊤}\phi_{\mathrm{ij}}\left({\hat{w}}_{j}\left(t-\tau \left(t\right)\right)-{\hat{w}}_{i}\left(t\right)\right)\\ \;-\sigma_{\mathrm{ij}}\text{sgn}{\left({\hat{v}}_{i}\left(t\right)-v_{i}\left(t\right)\right)}^{⊤}\phi_{\mathrm{ij}}\left({\hat{w}}_{j}\left(t-\tau \left(t\right)\right)-{\hat{w}}_{i}\left(t\right)\right) \end{array} \end{array}$$ (23c)

$$\begin{array}{c} d_{i}^{w}\left(t\right)=k_{i}^{w}{\left({\hat{w}}_{i}\left(t\right)-w_{i}\left(t\right)\right)}^{⊤}\left({\hat{w}}_{i}\left(t\right)-w_{i}\left(t\right)\right)\\ \;-\alpha \sqrt{k_{i}^{w}}\text{sgn}\left(d_{i}^{w}\left(t\right)-d^{*}\right) \end{array}$$ (23d)

and

$$\begin{array}{c} \left\{\begin{array}{c} {\dot{d}}_{i}^{v}\left(t\right)=k_{i}^{v}{\left({\hat{v}}_{i}\left(t\right)-v_{i}\left(t\right)\right)}^{⊤}\left({\hat{v}}_{i}\left(t\right)-v_{i}\left(t\right)\right)-\alpha \sqrt{k_{i}^{v}}\text{sgn}\left(d_{i}^{v}\left(t\right)-d^{*}\right)\\ \;-\frac{k_{i}^{v}\left(d_{i}^{v}\left(t\right)-d^{*}\right)}{{\left(d_{i}^{v}\left(t\right)-d^{*}\right)}^{2}}\left(\Gamma_{1}+\Gamma_{2}+\Gamma_{3}\right),\text{if }{\text{d}}_{i}^{v}\left(t\right){\text{≠d}}^{*}\text{,}\;\\ {\dot{d}}_{i}^{v}\left(t\right)=k_{i}^{v}{\left({\hat{v}}_{i}\left(t\right)-v_{i}\left(t\right)\right)}^{⊤}\left({\hat{v}}_{i}\left(t\right)-v_{i}\left(t\right)\right),\text{if}{\text{ d}}_{i}^{v}\left(t\right)\text{=}{\text{d}}^{*}\text{,} \end{array}\right. \end{array}$$ (23e)

where *i* = 1, …, *n*, $j\in N_{i},d^{*}$ is a sufficiently large positive number, *k_i_^w^*, *k_i_^v^*, *σ_ij_*, and *α* are positive constants, Γ_1_, Γ_2_, and Γ_3_ are given by:

$$\begin{array}{c} \Gamma_{1}=\sum_{j\in N_{i}}{\left\|(|a_{\mathrm{ij}}\left(t\right)|+\xi )\phi_{\mathrm{ij}}\left({\hat{x}}_{j}\left(t-\tau \left(t\right)\right)-{\hat{x}}_{i}\left(t\right)\right)\right\|}_{1}, \end{array}$$ (24)

$$\Gamma_{2}=\alpha \;\sum_{j\in N_{i}}\left(\frac{{\hat{a}}_{\mathrm{ij}}\left(t\right)|+\xi }{\sqrt{\sigma_{\mathrm{ij}}}}\right),$$ (25)

and

$$\Gamma_{3}=\alpha r{\left(\int_{t-\tau (t)}^{t}\sum_{i=1}^{N}e_{i}^{T}\left(s\right)e_{i}\left(s\right)ds\right)}^{\frac{1}{2}},$$ (26)

respectively, where *r* is a large positive constant.

Denoting $e_{i}^{w}\left(t\right)={\hat{w}}_{i}\left(t\right)-w_{i}\left(t\right)$, $e_{i}^{v}\left(t\right)={\hat{v}}_{i}\left(t\right)-v_{i}\left(t\right)$, and ${\tilde{a}}_{\mathrm{ij}}={\hat{a}}_{\mathrm{ij}}-a_{\mathrm{ij}}$, we can obtain the following the error system:

$$\begin{array}{c} \left\{\begin{array}{c} {\overset{\cdot }{e}}_{i}^{w}\left(t\right)=e_{i}^{v}\left(t\right)-d_{i}^{w}\left(t\right)\left({\hat{w}}_{i}\left(t\right)-w_{i}\left(t\right)\right)-\alpha \text{sgn}\left({\hat{w}}_{i}\left(t\right)-w_{i}\left(t\right)\right),\\ {\overset{\cdot }{e}}_{i}^{v}\left(t\right)=f\left(t,{\hat{v}}_{i}\left(t\right)\right)-f\left(t,v_{i}\left(t\right)\right)-d_{i}^{v}\left(t\right)\left({\hat{v}}_{i}\left(t\right)-v_{i}\left(t\right)\right)-\alpha \text{sgn}\left({\hat{v}}_{i}\left(t\right)-v_{i}\left(t\right)\right)\\ +\sum_{j\in N_{i}}{\hat{a}}_{\mathrm{ij}}\phi_{\mathrm{ij}}\left({\hat{w}}_{j}\left(t-\tau \left(t\right)\right)-{\hat{w}}_{i}\left(t\right)\right)-\sum_{j\in N_{i}}a_{\mathrm{ij}}\phi_{\mathrm{ij}}\left(w_{j}\left(t-\tau \left(t\right)\right)-w_{i}\left(t\right)\right). \end{array}\right. \end{array}$$ (27)

### Convergence analysis

**Theorem 4.1**. Suppose that Assumptions 2.1 and 2.2 hold. Then, the adjacency matrix *A* of the complex network (Eq. 21) can be identified by the proposed distributed observer (Eqs. 22 and 23a to 23e) in finite-time *t_n_* under any conditions, where *t_n_* is the upper bound of the convergence time, given by:

$$t_{n}=\frac{\sqrt{2V_{n}^{1/2}\left(0\right)}}{\alpha },$$ (28)

and *V_n_*(0) is given by:

$$\begin{array}{c} \begin{array}{l} V_{n}\left(0\right)=\frac{1}{2}\sum_{i=1}^{N}e_{i}^{w⊤}\left(0\right)e_{i}^{w}\left(0\right)+\frac{1}{2}\sum_{i=1}^{N}e_{i}^{v⊤}\left(0\right)e_{i}^{v}\left(0\right)+\frac{1}{2}\sum_{i=1}^{N}\sum_{j\in N_{i}}\frac{1}{\sigma_{\mathrm{ij}}}{\tilde{a}}_{\mathrm{ij}}^{2}\left(0\right)\\ \;+\frac{1}{2}\sum_{i=1}^{N}\frac{1}{k_{i}}{\left(d_{i}^{w}\left(0\right)-d^{*}\right)}^{2}+\frac{1}{2}r\int_{-\tau \left(0\right)}^{0}\sum_{i=1}^{N}e_{i}^{⊤}\left(s\right)e_{i}\left(s\right)\mathrm{ds}. \end{array} \end{array}$$ (29)

**Proof:** Construct a Lyapunov function *V_n_* as follows:

Vn(t)=12∑i=1Neiw⊤(t)+12∑i=1Neiv⊤(t)eiv(t)+12∑i=1N∑j∈Ni1σija˜ij2(t)+12∑i=1N1ki(diw(t)−d*)2+12∑i=1N1ki(div(t)−d*)2+12r∫t−τ(t)t∑i=1Nei⊤(s)ei(s)ds (30)

Taking the derivative of *V_n_* and substituting Eqs. 23a to 23e and 27 to ${\overset{\cdot }{V}}_{n}$, we can obtain

$${\overset{\cdot }{V}}_{n}\left(t\right)=W_{1}\left(t\right)+W_{2}\left(t\right),$$ (31)

where

W1(t)=∑i=1Neiw⊤(t)eiv(t)−d*∑i=1Neiw⊤(t)eiw(t)+∑i=1Neiv(t)(f(t,v^i(t))−f(t,vi(t)))−d*∑i=1Neiv⊤(t)eiv(t)+∑i=1N∑j∈Nieiv⊤(t)aijϕij(w^j(t−τ(t))−w^i(t))−∑i=1N∑j∈Nieiv⊤(t)aijϕij(wj(t−τ(t))−wi(t))+12r∑i=1Neiw⊤(t)eiw(t)−r2(1−τ·(t))∑i=1Neiw⊤(t−τ(t))eiw(t−τ(t)) (32)

and

$$\begin{array}{c} \begin{array}{l} W_{2}\left(t\right)=-\;\sum_{i=1}^{N}\sum_{j\in N_{i}}{\tilde{a}}_{\mathrm{ij}}\text{sgn}\left(e_{i}^{v⊤}\left(t\right)\right)\phi_{\mathrm{ij}}\left({\hat{w}}_{j}\left(t-\tau \left(t\right)\right)-{\hat{w}}_{i}\left(t\right)\right)\\ \;\_\sum_{i=1}^{N}\sum_{j\in N_{i}}{\left\|\left(|{\hat{a}}_{\mathrm{ij}}|+\xi \right)\phi_{\mathrm{ij}}\left({\hat{w}}_{j}\left(t-\tau \left(t\right)\right)-{\hat{w}}_{i}\left(t\right)\right)\right\|}_{1}-\alpha \;\sum_{i=1}^{N}e_{i}^{w⊤}\left(t\right)\text{sgn}\left(e_{i}^{w}\left(t\right)\right)\\ \;-\alpha \;\sum_{i=1}^{N}e_{i}^{v⊤}\left(t\right)\text{sgn}\left(e_{i}^{v}\left(t\right)\right)-\alpha \;\sum_{i=1}^{N}\left|\frac{d_{i}^{w}\left(t\right)-d^{*})}{\sqrt{k_{i}^{w}}}\right|-\alpha \;\sum_{i=1}^{N}\left|\frac{\left(d_{i}^{v}\left(t\right)-d^{*}\right)}{\sqrt{k_{i}^{v}}}\right|\\ \;-\alpha \;\sum_{i=1}^{N}\sum_{j\in N_{i}}\left(\frac{|{\hat{a}}_{\mathrm{ij}}|+\xi }{\sqrt{\sigma_{\mathrm{ij}}}}\right)-\frac{\alpha r}{2}{\left(\int_{t-\tau \left(t\right)}^{t}\sum_{i=1}^{N}e_{i}^{w⊤}\left(s\right)e_{i}^{w}\left(s\right)\mathrm{ds}\right)}^{\frac{1}{2}}, \end{array} \end{array}$$ (33)

respectively.

Denote $\begin{array}{c} w\left(t\right)={\left(w_{1}^{⊤}\left(t\right),w_{2}^{⊤}\left(t\right),\ldots ,w_{N}^{⊤}\left(t\right)\right)}^{⊤}\in {\mathbb{R}}^{\mathrm{nN}\times 1} \end{array}$, $\begin{array}{c} v(t)= \end{array}$$\begin{array}{c} v(t)= \end{array}\begin{array}{c} (v_{1}^{⊤}\left(t\right),v_{2}^{⊤}\left(t\right),\ldots ,v_{N}^{⊤}\left(t\right){)}^{⊤}\in {\mathbb{R}}^{\mathrm{nN}\times 1} \end{array}$,$\begin{array}{c} \hat{w}\left(t\right)=({\hat{w}}_{1}^{⊤}\left(t\right),{\hat{w}}_{2}^{⊤}\left(t\right),\ldots , \end{array}$, $\begin{array}{c} {\hat{w}}_{N}^{⊤}\left(t\right){)}^{⊤}\in {\mathbb{R}}^{\mathrm{nN}\times 1} \end{array}$, $\hat{v}\left(t\right)={\left({\hat{v}}_{1}^{⊤}\left(t\right),{\hat{v}}_{2}^{⊤}\left(t\right),\ldots ,{\hat{v}}_{N}^{⊤}\left(t\right)\right)}^{⊤}\in {\mathbb{R}}^{\mathrm{nN}\times 1}$, $\begin{array}{c} e^{w}\left(t\right)={\left(e_{1}^{w⊤}\left(t\right),e_{2}^{w⊤}\left(t\right),\ldots ,e_{N}^{w⊤}\left(t\right)\right)}^{⊤}\in {\mathbb{R}}^{\mathrm{nN}\times 1} \end{array}$, $\begin{array}{c} e^{v}(t)=(e_{1}^{v⊤}(t), \end{array}$$\begin{array}{c} e^{v}\left(t\right)=(e_{1}^{v⊤}\left(t\right), \end{array}\begin{array}{c} e_{2}^{v⊤}\left(t\right),\ldots ,e_{N}^{v⊤}\left(t\right){)}^{⊤}\in {\mathbb{R}}^{\mathrm{nN}\times 1} \end{array}$, and $\begin{array}{c} \Phi w(t)=[\Phi_{1}^{⊤}(w),\Phi_{2}^{⊤}(w),\ldots , \end{array}$ $\begin{array}{c} \Phi \left(w\right)=[\Phi_{1}^{⊤}(w),\Phi_{2}^{⊤}(w),\ldots , \end{array}\begin{array}{c} \Phi_{N}^{⊤}\left(w\right){]}^{⊤}\in {\mathbb{R}}^{\mathrm{nN}\times 1} \end{array}$,where Φ(*w*) ∈ *ℝ*^*nN*×1^, and

$$\begin{array}{c} \Phi i\left(w\left(t-\tau \left(t\right)\right)\right)=\;\sum_{j\in N_{i}}a_{\mathrm{ij}}\phi_{\mathrm{ij}}(w_{j}\left(t-\tau \left(t\right)\right)-w_{i}\left(t\right)). \end{array}$$ (34)

Then, *W*_1_(*t*) can be written as

W1(t)=ew⊤(t)ev(t)−d*ew⊤(t)ew(t)+ev⊤(t)(f(t,v^(t))−f(t,v(t)))−d*ev⊤(t)ev(t)+ev⊤[Φ(w^(t−τ(t)))−Φ(w(t−τ(t)))]+12r∑i=1Neiw⊤(t)eiw(t)−r2(1−τ·(t))∑i=1Neiw⊤(t−τ(t))eiw(t−τ(t)) (35)

where $\begin{array}{c} (f(t,v(t))=f(t,v_{⊤}^{1}(t)),f(t,v_{1}^{⊤}(t)),\ldots f(t,v_{N}^{⊤}(t)){)}^{⊤}\in {\mathbb{R}}^{nN\times 1} \end{array}$.

According to Assumption 2.1, we can obtain that there exist constants *L*_1_ and *L*_2_ satisfying the following conditions:

$$\begin{array}{c} \left\|f\left(t,\hat{v}\left(t\right)\right)-f\left(t,v\left(t\right)\right)\|\leq L_{1}\|\hat{v}\left(t\right)-v\left(t\right)\right\| \end{array}$$ (36)

and

$$\begin{array}{c} \left\|\Phi \left(\hat{w}\left(t-\tau \left(t\right)\right)\right)-\Phi \left(w\left(t-\tau \right)\right))\right\|\\ \leq L_{2}\|\hat{w}\left(t-\tau \left(t\right)\right)-w\left(t-\tau \left(t\right)\right)\|. \end{array}$$ (37)

Then, according to Lemma 2.1, *W*_1_(*t*) can be written as

$$\begin{array}{c} \begin{array}{l} W_{1}\left(t\right)\leq \frac{1}{2}e^{w⊤}\left(t\right)e^{w}\left(t\right)+\frac{1}{2}e^{v⊤}\left(t\right)e^{v}\left(t\right)-d^{*}e^{w⊤}\left(t\right)e^{w}\left(t\right)+L_{1}e^{v⊤}\left(t\right)e^{v}\left(t\right)-d^{*}e^{v⊤}\left(t\right)e^{v}\left(t\right)\\ \;+\frac{L_{2}}{2}e^{v⊤}\left(t\right)e^{v}\left(t\right)+\frac{L_{2}}{2}e^{w⊤}\left(t-\tau \left(t\right)\right)e^{w}\left(t-\tau \left(t\right)\right)+\frac{1}{2}{\mathrm{re}}^{w⊤}\left(t\right)e^{w}\left(t\right)\\ \;-\frac{r}{2}\left(1-\overset{\cdot }{\tau }\left(t\right)\right)e^{w⊤}\left(t-\tau \left(t\right)\right)e^{w}\left(t-\tau \left(t\right)\right)\\ \;\leq \left(\frac{1}{2}+\frac{1}{2}r-d^{*}\right)e^{w⊤}\left(t\right)e^{w}\left(t\right)+\left(\frac{L_{2}}{2}-\frac{r}{2}\left(1-\overset{\cdot }{\tau }\left(t\right)\right)\right)e^{w⊤}\left(t-\tau \left(t\right)\right)e^{w}\left(t-\tau \left(t\right)\right)\\ \;+\left(\frac{1}{2}+L_{1}+\frac{L_{2}}{2}-d^{*}\right)e^{v⊤}\left(t\right)e^{v}\left(t\right) \end{array} \end{array}$$ (38)

Let $\begin{array}{c} r\geq \frac{L_{2}}{1-\overset{\cdot }{\tau }\left(t\right)} \end{array}$ and $d^{*}\geq \text{max}\left\{\frac{1}{2}\left(1+r\right),\frac{1}{2}\left(1+L_{2}\right)+L_{1}\right\}$, we can derive that *W*_1_(*t*) ≤ 0 and $\begin{array}{c} {\overset{\cdot }{V}}_{n}\left(t\right)\leq W_{2}\left(t\right) \end{array}$.

Regarding *W*_2_(*t*), we can leverage the inherent properties of the sign function to obtain that

$$\begin{array}{c} \begin{array}{l} W_{2}\left(t\right)\leq \sum_{i=1}^{N}\sum_{j\in N_{i}}{\left\|{\tilde{a}}_{\mathrm{ij}}\phi_{\mathrm{ij}}\left({\hat{w}}_{j}\left(t-\tau \left(t\right)\right)-{\hat{w}}_{j}\left(t\right)\right)\right\|}_{1}-\sum_{i=1}^{N}\\ \;\sum_{j\in N_{i}}{\left\|\left(\left|{\hat{a}}_{\mathrm{ij}}\right|+\xi \right)\phi_{\mathrm{ij}}\left({\hat{w}}_{j}\left(t-\tau \left(t\right)\right)-{\hat{w}}_{i}\left(t\right)\right)\right\|}_{1}\\ \;-\alpha \;\sum_{i=1}^{N}\left\|e_{i}^{w},\left(t\right)\right\|-\alpha \;\sum_{i=1}^{N}\left\|e_{i}^{v},\left(t\right)\right\|-\alpha \sum_{i=1}^{N}\left|\frac{\left(d_{i}^{w}\left(t\right)-d^{*}\right)}{\sqrt{k_{i}^{w}}}\right|-\alpha \;\sum_{i=1}^{N}\left|\frac{\left(d_{i}^{v}\left(t\right)-d^{*}\right)}{\sqrt{k_{i}^{v}}}\right|\\ \;-\alpha \;\sum_{i=1}^{N}\sum_{j\in N_{i}}\left(\frac{|{\hat{a}}_{\mathrm{ij}}|+\xi }{\sqrt{\sigma_{\mathrm{ij}}}}\right)-\frac{\alpha r}{2}{\left(\int_{t-\tau \left(t\right)}^{t}\sum_{i=1}^{N}e_{i}^{w⊤}\left(s\right)e_{i}^{w}\left(s\right)\mathrm{ds}\right)}^{\frac{1}{2}}. \end{array} \end{array}$$ (39)

According to Eq. 15, *W*_2_(*t*) can be written as

$$\begin{array}{c} \begin{array}{l} W_{2}\left(t\right)\leq -\alpha \;\sum_{i=1}^{N}\left\|e_{i}^{w},\left(t\right)\right\|-\alpha \;\sum_{i=1}^{N}\left\|e_{i}^{v},\left(t\right)\right\|-\alpha \;\sum_{i=1}^{N}\left|\frac{\left(d_{i}^{w}\left(t\right)-d^{*}\right)}{\sqrt{k_{i}^{w}}}\right|-\alpha \;\sum_{i=1}^{N}\left|\frac{\left(d_{i}^{v}\left(t\right)-d^{*}\right)}{\sqrt{k_{i}^{v}}}\right|\\ \;-\alpha \;\sum_{i=1}^{N}\sum_{j\in N_{i}}\left(\frac{|{\hat{a}}_{\mathrm{ij}}|+\xi }{\sqrt{\sigma_{\mathrm{ij}}}}\right)-\frac{\alpha r}{2}{\left(\int_{t-\tau \left(t\right)}^{t}\sum_{i=1}^{N}e_{i}^{w⊤}\left(s\right)e_{i}^{w}\left(s\right)d\;s\right)}^{\frac{1}{2}}. \end{array} \end{array}$$ (40)

Finally, according to Lemma 2.2, we can obtain that

$$\begin{array}{c} {\dot{V}}_{n}\left(t\right)\leq -\sqrt{2\alpha }\left[\frac{1}{2}\sum_{i=1}^{N}e_{i}^{w⊤}\left(t\right)e_{i}^{w}\left(t\right)+\frac{1}{2}\sum_{i=1}^{N}e_{i}^{v⊤}\left(t\right)e_{i}^{v}\left(t\right)+\frac{1}{2}\sum_{i=1}^{N}\frac{1}{k_{i}}{\left(d_{i}^{w}\left(t\right)-d^{*}\right)}^{2}\right.\\ {\left.+\frac{1}{2}\sum_{i=1}^{N}\frac{1}{k_{i}}{\left(d_{i}^{v}\left(t\right)-d^{*}\right)}^{2}+\frac{1}{2}\sum_{i=1}^{N}\sum_{j\in N_{i}}\frac{1}{\sigma_{\mathrm{ij}}}{\tilde{a}}_{\mathrm{ij}}^{2}+\frac{1}{2}r\int_{t-\tau \left(t\right)}^{t}\sum_{i=1}^{N}e_{i}^{⊤}\left(s\right)e_{i}\left(s\right)\mathrm{ds}\right]}^{\frac{1}{2}} \end{array}$$ (41)

According to Lemma 2.3, when $\begin{array}{c} t\geq t_{n}=\frac{\sqrt{2}V_{n}^{1/2}\left(0\right)}{\alpha } \end{array}$, *V_n_*(*t*) = 0, the drive network (Eq. 21) and the response network (Eq. 22) achieve finite-time synchronization, and the uncertain parameters in the drive network can be correctly identified. From Eq. 30, it can obtained that $\begin{array}{c} \underset{t\to t_{n}}{\text{lim}}\;\left\|e_{i}^{w}\left(t\right)\right\|=0 \end{array}$, $\begin{array}{c} \underset{t\to t_{n}}{\text{lim}}\;\left\|e_{i}^{v}\left(t\right)\right\|=0 \end{array}$, $\underset{t\to t_{n}}{\text{lim}}|{\tilde{a}}_{\mathrm{ij}}\left(t\right)|=0$, $\underset{t\to t_{n}}{\text{lim}}|d_{i}^{w}\left(t\right)-d^{*}|=0$, and $\underset{t\to t_{n}}{\text{lim}}|d_{i}^{v}\left(t\right)-d^{*}|=0$.

### Distributed finite-time observer for power grids

In this subsection, we propose a distributed finite-time observer for the power grid to identify the variation in network topology.

We utilize an undirected graph G=(N, Ω) to represent the power grid, where $N=\{1,2,\ldots ,n_{s}\}$ represents the bus set and $\Omega =\{(i,j),i\in N,j\in N_{i}\}$ represents the set of all transmission lines in the power grid, while $N_{i}$ represents the set of buses connected to bus *i*. *N* = $N_{g}\cup N_{i}.N_{g}$and $N_{i}$ denote the sets of generator buses and load buses, respectively, where bus *i* ∈ $N_{g}$ if *i* = 1,2, ⋯, *n*_g_ and *i* ∈ $N_{i}$ if *i* = *n*_g_ + 1, *n*_g_ + 2, ⋯, *n_s_*.

On the basis of [31], the power grid can be formulated as follows

$$\begin{array}{c} \left\{\begin{array}{c} {\dot{\theta }}_{i}=w_{i},\\ {\dot{w}}_{i}=\frac{1}{M_{i}}\left(P_{m,i}-D_{i}w_{i}\right)+\sum_{j\in N_{i}}B_{\mathrm{ij}}l_{\mathrm{ij}}\left(\theta_{i}-\theta_{j}\right),i\in N_{g} \end{array}\right. \end{array}$$ (42a)

$$\begin{array}{c} {\overset{\cdot }{\theta }}_{i}=-\;\frac{1}{D_{i}}P_{d,i}+\sum_{j\in N_{i}}B_{\mathrm{ij}}l_{\mathrm{ij}}\left(\theta_{i}-\theta_{j}\right),i\in N_{l} \end{array}$$ (42b)

where *V_i_* and *θ_i_* represent the voltage magnitude and angle at bus *i*, respectively. *ω_i_* indicates the frequency relative to the nominal frequency of generator bus *i*. *P*_*m*,*i*_ represents the mechanical power supplied by the generator bus *i*, and *P*_*d*,*i*_ denotes the active power requested by the load bus *i*. In addition, *M_i_* represents the inverse of the rotor inertia, and *D_i_* stands for the damping ratio. The value *B_ij_* signifies the imaginary part of the admittance of transmission lines (*i*, *j*). Moreover, the specific expression *l_ij_*(.) of the function mentioned in (Eqs. 42a and 42b is as follows

$$\begin{array}{c} l_{\mathrm{ij}}\left(\theta_{i}-\theta_{j}\right)=\left\{\begin{array}{c} -\frac{1}{M_{i}}V_{i}V_{j}\sin\left(\theta_{i}-\theta_{j}\right),i\in N_{g},\;\\ -\frac{1}{D_{i}}V_{i}V_{j}\sin\left(\theta_{i}-\theta_{j}\right),i\in N_{l},\; \end{array}\right. \end{array}$$

Then, according to Theorem 4.1, we design an observer (Eqs. 43a to 43f) to identify the topology of the dynamical power grid (Eqs. 42a and 42b), which can converge within a finite time.

$$\begin{array}{c} \left\{\begin{array}{c} {\dot{\hat{\theta }}}_{i}={\hat{w}}_{i}-d_{i}^{\theta }\left({\hat{\theta }}_{i}-\theta_{i}\right)-\alpha \text{sgn}\left({\hat{\theta }}_{i}-\theta_{i}\right),\\ {\dot{\hat{w}}}_{i}=\frac{1}{M_{i}}\left(P_{m,i}-D_{i}{\hat{w}}_{i}\right)+\sum_{j\in N_{i}}{\hat{B}}_{\mathrm{ij}}l_{\mathrm{ij}}\left({\hat{\theta }}_{i}-\theta_{j}\right)-d_{i}^{w}\left({\hat{w}}_{i}-w_{i}\right)-\alpha \text{sgn}\left({\hat{w}}_{i}-w_{i}\right),i\in N_{g} \end{array}\right., \end{array}$$ (43a)

$$\begin{array}{c} {\dot{\hat{\theta }}}_{i}=-\frac{1}{D_{i}}P_{d,i}+\sum_{j\in N_{i}}{\hat{B}}_{\mathrm{ij}}l_{\mathrm{ij}}\left({\hat{\theta }}_{i}-\theta_{j}\right)-d_{i}^{\theta }\left({\hat{\theta }}_{i}-\theta_{i}\right)-\alpha \text{sgn}\left({\hat{\theta }}_{i}-\theta_{i}\right),i\in N_{l},\\ \; \end{array}$$ (43b)

$$\begin{array}{c} {\overset{\cdot }{\hat{B}}}_{\mathrm{ij}}=-\;\sigma_{\mathrm{ij}}^{B}\left(\text{sgn}\left({\hat{w}}_{i}-w_{i}\right)+\left({\hat{w}}_{i}-w_{i}\right)\right)l_{\mathrm{ij}}\left({\hat{\theta }}_{i}-{\hat{\theta }}_{j}\right),i\in N_{g},,i\in N_{i}, \end{array}$$ (43c)

$$\begin{array}{c} {\overset{\cdot }{\hat{B}}}_{\mathrm{ij}}=-\;\sigma_{\mathrm{ij}}^{B}\left(\text{sgn}\left({\hat{\theta }}_{i}-\theta_{i}\right)+\left({\hat{\theta }}_{i}-{\hat{\theta }}_{i}\right)\right)l_{\mathrm{ij}}\left({\hat{\theta }}_{i}-{\hat{\theta }}_{j}\right),i\in N_{g},,i\in N_{i}, \end{array}$$ (43d)

$$\begin{array}{c} \left\{\begin{array}{c} {\dot{d}}_{i}^{\theta }=\sigma_{i}^{\theta }{\left({\hat{\theta }}_{i}-\theta_{i}\right)}^{2}-\alpha \sqrt{\sigma_{i}^{\theta }}\mathrm{sgn}\left(d_{i}^{\theta }-d^{*}\right)\\ {\dot{d}}_{i}^{w}=\sigma_{i}^{w}{\left({\hat{w}}_{i}-w_{i}\right)}^{2}-\alpha \sqrt{\sigma_{i}^{w}}\mathrm{sgn}\left(d_{i}^{w}-d^{*}\right)-\frac{\sigma_{i}^{w}}{d_{i}^{w}-d^{*}}\left(\Theta_{1}+\Theta_{2}\right),\text{if}\;d_{i}^{w}\neq d^{*}\\ {\dot{d}}_{i}^{w}=\sigma_{i}^{w}{\left({\hat{w}}_{i}-w_{i}\right)}^{2},\text{if}\;d_{i}^{w}=d^{*},i\in N_{g} \end{array}\right., \end{array}$$ (43e)

and

$$\begin{array}{c} \left\{\begin{array}{c} {\dot{d}}_{i}^{\theta }=\sigma_{i}^{\theta }{\left({\hat{\theta }}_{i}-\theta_{i}\right)}^{2}-\alpha \sqrt{\sigma_{i}^{\theta }}\mathrm{sgn}\left(d_{i}^{\theta }-d^{*}\right)-\frac{\sigma_{i}^{\theta }}{\left(d_{i}^{\theta }-d^{*}\right)}\left(\Theta_{1}+\Theta_{2}\right),\text{if}\;d_{i}^{\theta }\neq d^{*}\\ {\dot{d}}_{i}^{\theta }=\sigma_{i}^{\theta }{\left({\hat{\theta }}_{i}-\theta_{i}\right)}^{2},\text{if}\;d_{i}^{\theta }\neq d^{*},i\in N_{l} \end{array}\right., \end{array}$$ (43f)

where $\begin{array}{c} {\hat{\theta }}_{i} \end{array}$, $\begin{array}{c} {\hat{\omega }}_{i} \end{array}$, and $\begin{array}{c} {\hat{B}}_{\mathrm{ij}} \end{array}$ are the observed values of *θ_i_*, *ω_i_*, and *B_ij_*, respectively. *α* is a positive constant, *α* > 0. *d_i_^ω^* and *d_i_^θ^* are the adaptive feedback gains, $d^{*}$ is a sufficiently large positive constant. *k_i_^ω^*, *k_i_^θ^*, and *σ_ij_^B^* are arbitrary positive constants, Θ_1_ and Θ_2_ are given by:

$$\Theta_{1}=\sum_{j\in N_{i}}|\left(|{\hat{B}}_{\mathrm{ij}}|+\xi \right)l_{\mathrm{ij}}\left({\hat{\theta }}_{i}-{\hat{\theta }}_{j}\right)|,$$ (44)

and

$$\Theta_{2}=\alpha \sum_{j\in N_{i}}\left(\frac{\left|{\hat{B}}_{ij}\right|+\xi }{\sqrt{\sigma_{ij}^{B}}}\right)\;$$ (45)

respectively, where *ξ* is a positive constant, given by:

$$\underset{i\in N,j\in N_{i}}{\text{max}}|B_{\mathrm{ij}}|\leq \xi .$$ (46)

Then, the finite-time line-outage detection algorithm is proposed here. Once the observation system reaches a stable state, any large change in the observed admittance values is considered an indication of line outage. To enhance the detection speed, we introduce a detection threshold denoted as Δ. If $|{\hat{B}}_{\mathrm{ij}}|\leq \Delta B_{\mathrm{ij}}$ and (*i*, *j*) ∈ Ω, the transmission line (*i*, *j*) cuts off.

**Remark 4.1**. The use of distributed computing is used here. Each node update its corresponding distributed observer using its own local measurements as well as information from neighboring nodes.

**Remark 4.2**. The existing finite-time observers [25–27] designed for topology identification of complex networks mainly rely on the difference between the true values of edge weights and their observed values. In the case of applying these observers to power grids, it is essential to acquire the true admittance values, which is not feasible in the field of power grid topology identification. However, our proposed finite-time observer can achieve topology identification without the need to obtain the true values of the admittance.

## Results and Discussion

We perform numerical experiments on both complex networks and power grids to comprehensively evaluate the effectiveness of the proposed finite-time topology identification method.

Case 1: Consider a complex dynamical network consisting of 15 nodes, where the first 5 nodes are Lorenz systems and the remaining nodes are Chen systems, given by

$$\begin{array}{c} f_{i}(x_{i})=\left[\begin{array}{c} 10\left(x_{i2}-x_{i1}\right)\\ 28x_{i1}-x_{i1}x_{i3}-x_{i2}\\ x_{i1}x_{i2}-8/3x_{i3} \end{array}\right],i=1,2,\ldots ,5, \end{array}$$ (47)

and

$$\begin{array}{c} f_{i}(x_{i})=\left[\begin{array}{c} 35\left(x_{i2}-x_{i1}\right)\\ -7x_{i1}-x_{i1}x_{i3}+28x_{i2}\\ x_{i1}x_{i2}-3x_{i3} \end{array}\right],i=6,7,\ldots ,15, \end{array}$$ (48)

respectively. The initial values of state variables are randomly selected from [−2,2]. The weighted configuration matrix *A* is described as follows

$$\begin{array}{c} A=\left[\begin{array}{cccccccccc} -10 & 2 & 0 & 0 & 0 & 1 & 4 & 0 & 0 & 0\\ 1 & 0 & 0 & 0 & 2 & & & & & \\ 2 & -13 & 0 & 5 & 0 & 3 & 0 & 0 & 1 & 0\\ 0 & 0 & 0 & 2 & 0 & & & & & \\ 0 & 0 & -10 & 2 & 0 & 0 & 0 & 0 & 4 & 4\\ 0 & 0 & 0 & 0 & 0 & & & & & \\ 0 & 5 & 2 & -13 & 0 & 0 & 0 & 0 & 0 & 0\\ 6 & 0 & 0 & 0 & 0 & & & & & \\ 0 & 0 & 0 & 0 & -11 & 0 & 2 & 2 & 0 & 0\\ 7 & 0 & 0 & 0 & 0 & & & & & \\ 1 & 3 & 0 & 0 & 0 & -9 & 0 & 4 & 0 & 0\\ 0 & 0 & 0 & 0 & 1 & & & & & \\ 4 & 0 & 0 & 0 & 2 & 0 & -9 & 0 & 0 & 0\\ 0 & 0 & 3 & 0 & 0 & & & & & \\ 0 & 0 & 0 & 0 & 2 & 4 & 0 & -8 & 0 & 0\\ 0 & 2 & 0 & 0 & 0 & & & & & \\ 0 & 1 & 4 & 0 & 0 & 0 & 0 & 0 & -6 & 1\\ 0 & 0 & 0 & 0 & 0 & & & & & \\ 0 & 0 & 4 & 0 & 0 & 0 & 0 & 0 & 1 & -14\\ 9 & 0 & 0 & 0 & 0 & & & & & \\ 1 & 0 & 0 & 6 & 7 & 0 & 0 & 0 & 0 & 9\\ -23 & 0 & 0 & 0 & 0 & & & & & \\ 0 & 0 & 0 & 0 & 0 & 0 & 0 & 2 & 0 & 0\\ 0 & -8 & 6 & 0 & 0 & & & & & \\ 0 & 0 & 0 & 0 & 0 & 0 & 3 & 0 & 0 & 0\\ 0 & 6 & -9 & 0 & 0 & & & & & \\ 0 & 2 & 0 & 0 & 0 & 0 & 0 & 0 & 0 & 0\\ 0 & 0 & 0 & -6 & 4 & & & & & \\ 2 & 0 & 0 & 0 & 0 & 1 & 0 & 0 & 0 & 0\\ 0 & 0 & 0 & 4 & -7 & & & & & \end{array}\right]. \end{array}$$ (49)

The initial values of $\begin{array}{c} {\hat{a}}_{\mathrm{ij}} \end{array}$ and *d_i_* are randomly chosen from [−10,10]. The gains in Eqs. 4a to 4c are taken as *k_i_* = *δ_ij_* = 10, and let $\begin{array}{c} d^{*} \end{array}$ = 200, *α* = 2, and *ξ* = 20. The inner coupling matrix *H* between nodes is taken as the identity matrix of order 3. The time unit regarding *t* and time delay *τ*(*t*) is the second (the iterations of ode45), *τ*(*t*) = 1.

**Fig. 2.**
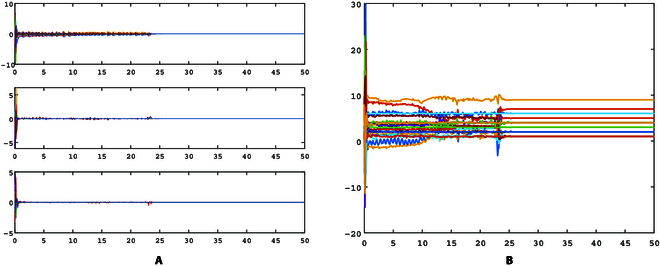
Identification errors of delayed network (Eq. 2) with adaptive observation approach. (A) Generalized synchronization errors. (B) Topology identification results.

**Fig. 3.**
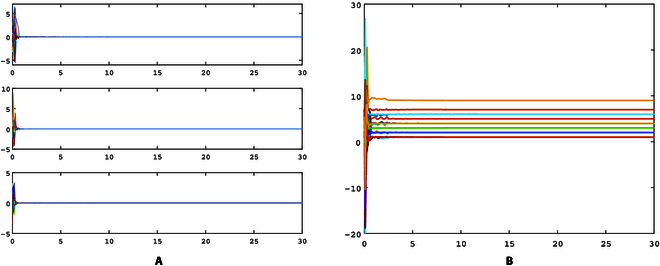
Identification errors of delayed network (Eq. 2) with finite-time observation approach. (A) Generalized synchronization errors. (B) Topology identification results.

Figure 2 illustrates the synchronization error and topology identification results of the conventional adaptive method, and Fig. 3 illustrates the synchronization error and topology identification results of the proposed finite-time method. Obviously, the identification speed of the adaptive method is significantly slower than the finite-time method. The adaptive identification method identifies all the topology weights at roughly *t* = 25.7 s, while the proposed finite-time identification method achieves it at *t* = 4.7 s. It is evident that the proposed finite-time method can significantly reduce the identification time and improve the synchronization speed.

Case 2: The proposed finite-time detection algorithm is tested with IEEE 39-bus system.

During the simulation, the initial state variables are randomly selected from [ − 10,10], and the initial topology observed values $\begin{array}{c} {\hat{B}}_{\mathrm{ij}} \end{array}$ are all selected from [0,250]. Define *M_i_* = *D_i_* = 1. The feedback gains *σ_i_^θ^* = *σ_i_^ω^* = 10, *σ_ij_^B^* = 200, and d* = 1000. Let *α* = 2, *ξ* = 400, and Δ = 0.25.

**Fig. 4.**
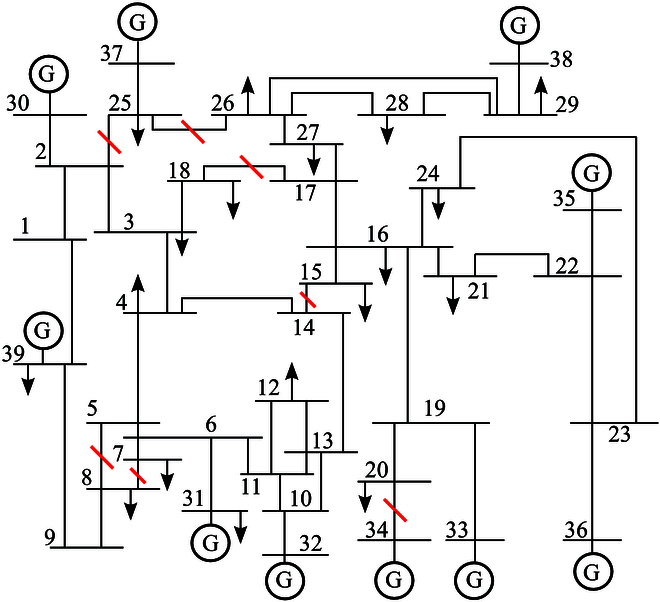
Location of line outages in IEEE 39-bus system.

Assuming that lines (2,25), (7,8), and (14,15) cut off at *t* = 80 s (the iterations of ode45), lines (5,8), (17,18), (20,34), and (25,26) cut off at *t* = 120 s, the location of line outages is shown in Fig. 4. The actual admittance values of these outage lines are list in Table 1.

**Table 1.** The actual admittance values of outage lines in IEEE 39-bus system

Branch	(2,25)	(7,8)	(14,15)	(5,8)	(17,18)	(20,34)	(25,36)
*B_ij_*	69.9414	215.7598	45.7680	88.8325	121.0690	54.9227	30.6588

**Fig. 5.**
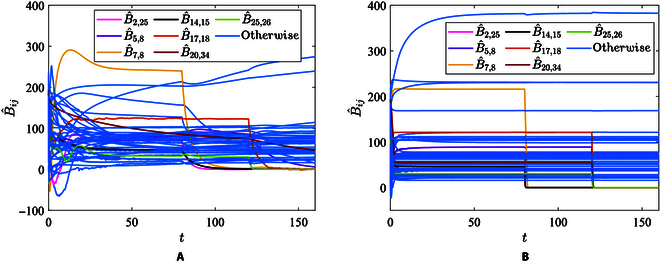
Comparison of line-outage detection results of the adaptive observer and finite-time observer in IEEE 39-bus system. (A) The observed admittance values of ${\hat{B}}_{\mathrm{ij}}$ by the adaptive observer [22]. (B) The observed admittance values of ${\hat{B}}_{\mathrm{ij}}$ by the finite-time observer.

The observed values of ${\hat{B}}_{\mathrm{ij}}$ with the adaptive method proposed in [22] and with the proposed finite-time method are shown in Fig. 5A and B, respectively. As shown in Fig. 5A, the adaptive observer has not yet achieved asymptotic convergence within 160 s. According to the detection threshold, the outage of lines (2,25), (7,8), and (14,15) can be detected at *t* = 84.6 s, *t* = 87.1 s, and *t* = 89.6 s, respectively. Similarly, the outage of lines (25,36), (17,18), and (5,8) can be detected at *t* = 121.6 s, *t* = 125.7 s, and *t* = 139.9 s, respectively, while the outage of line (20,34) can not be detected until *t* = 160 s. As shown in Fig. 5B, during the initialization process, the finite-time observer converges at *t* = 39.6 s. Obviously, the outage of lines (2,25), (7,8), and (14,15) can be detected at *t* = 80.4 s, *t* = 81.3 s, and *t* = 80.3 s, respectively. Similarly, the outage of lines (5,8), (17,18), (20,34), and (25,36) can be detected at *t* = 120.6 s, *t* = 120.8 s, *t* = 120.6 s, and *t* = 120.4 s, respectively. The observed admittance values of outage lines with the adaptive observer and the finite-time observer at different times are listed in Tables 2 and 3, respectively.

**Table 2.** The observed admittance values of ${\hat{B}}_{\mathrm{ij}}$ with the adaptive observer at different times

Time	*t* = 80 s	*t* = 81.3 s	*t* = 89.6 s	*t* = 120 s	*t* = 120.8 s	*t* = 139.9 s	*t* = 160 s
(2,25)	70.3476	45.7248	4.1240	−0.0430	0.7918	−0.0057	−0.0044
(7,8)	239.7425	166.5755	35.9141	2.2838	2.7424	3.2957	0.9768
(14,15)	46.7992	37.4903	11.3140	0.7754	0.7294	0.0185	0.1181
(5,8)	80.2717	85.9418	97.3993	85.8425	80.1421	22.1172	5.8897
(17,18)	123.7898	123.6103	122.3560	122.5421	90.5972	1.4658	1.0351
(20,34)	85.3114	84.7878	81.9597	74.0801	73.3405	58.4551	46.6320
(25,26)	32.1009	36.4090	31.6506	30.0252	18.8689	0.0106	−0.0153

**Table 3.** The observed admittance values of ${\hat{B}}_{\mathrm{ij}}$ with the finite-time observer at different times

Time	*t* = 80 s	*t* = 81.3 s	*t* = 89.6 s	*t* = 120 s	*t* = 120.8 s	*t* = 139.9 s	*t* = 160 s
(2,25)	69.9503	0.0202	−0.0014	−0.0020	−0.0010	−0.0032	−0.0010
(7,8)	215.7962	46.2957	−0.0011	−0.0007	−0.1204	−0.0003	−0.0004
(14,15)	45.7826	0.0309	−0.0015	−0.0009	−0.0039	−0.0004	−0.0006
(5,8)	88.8065	88.1459	88.7210	88.8202	5.1035	0.0036	0.0003
(17,18)	121.0436	121.1485	121.0678	121.0384	15.3863	−0.0004	0.0018
(20,34)	54.9210	54.9233	54.9233	54.9233	3.8832	0.0005	−0.0006
(25,26)	30.6453	30.9702	30.7040	30.7028	0.8568	0.0004	−0.0007

It is obvious that our proposed finite-time algorithm can detect line outages/the variation of edge weights within a finite time. Compared with the adaptive observer, the finite-time observer has a faster identification rate, the observed values of other normal lines are less affected, which means that our proposed algorithm has higher reliability. Furthermore, it is evident that the observer-based method can effectively solve the scenarios of successive line outages/topology changes.

## Conclusion

This paper addresses the problem of finite-time topology identification for complex networks with time delay. It introduces a novel method for finite-time observation, which is analytically proven to converge within a finite time. The method can also be applied to second-order dynamical networks with nonlinear coupling, which can also converge in finite time. In addition, a distributed finite-time detection algorithm has been designed on the basis of this observation method for power grids line-outage detection. Numerical experimental results demonstrate the superiority of the proposed finite-time observation method for fast and accurate topology identification. In future research, we will take the switch topology and cyber-attack into consideration.

## Data Availability

The data used to support the findings of this study are available from the corresponding author upon request.
